# Laparoscopic ureteroneocystostomy in the treatment of urinary incontinence due to ectopy of the ureters in female dogs: A pilot study

**DOI:** 10.1371/journal.pone.0292485

**Published:** 2023-10-05

**Authors:** Przemysław Prządka, Bartłomiej Liszka, Ludwika Gąsior, Agnieszka Antończyk, Piotr Skrzypczak, Zdzisław Kiełbowicz, Dominika Kubiak-Nowak, Sylwester Gerus, Dariusz Patkowski

**Affiliations:** 1 Department and Clinic of Surgery, Faculty of Veterinary Medicine, Wroclaw University of Environmental and Life Sciences, Wroclaw, Poland; 2 Department of Pediatric Surgery and Urology, Medical University of Wroclaw, Wroclaw, Poland; University of Catania, ITALY

## Abstract

Ureteral ectopia is rare and requires surgical treatment after a thorough diagnostic workup. Open surgical techniques for repositioning ectopic ureters have been known for many years and are well described in the literature. However, to the best of our knowledge, no laparoscopic method of correcting this pathology has been described, which, in our opinion, would benefit the animal in terms of the healing process and overall clinical outcomes. This study aimed to evaluate the possibility of laparoscopic treatment of ureteral ectopia, which causes urinary incontinence in dogs. All of the operated ten dogs presented in this study were client-owned females with symptoms of urinary incontinence due to a unilateral intramural ectopic ureter. A three-trocar laparoscopic technique was used to perform the ureteroneocystostomy of the ectopic ureter. In this article, clinicopathological data, imaging features, procedural findings, complications, and short- and long-term outcomes are presented. The procedure was feasible in all cases. No major postoperative complications were observed. Among the minor complications, slight hematuria was observed in three dogs, which resolved spontaneously. In the period of at least one year after surgery, no negative impact of the procedure was observed. Seven of the ten operated dogs regained urinary continence. The remaining three dogs required additional surgery (urethral bulking) because of a lack of improvement after adjuvant pharmacological treatment. Overall, good-to-excellent long-term outcomes can be achieved; however, dogs that remain incontinent after laparoscopic ureteroneocystostomy may require additional treatment.

## Introduction

Ectopic ureters (EU) are rare congenital malformations characterized by the opening of one or both ureteral orifices in a location other than the trigone of the urinary bladder [1]. This anomaly arises from the abnormal origin and migration of the ureteral bud along the mesonephric duct [2]. The presence of an ectopic ureter is often associated with other urinary tract anomalies, including renal aplasia, renal hypoplasia, ureterocele, hydronephrosis, hydroureter, ureterovesicular abnormalities, pelvic bladder, and urethral sphincter mechanism incompetence [1, 2]. The clinical presentation of dogs with ectopic ureters is most often related to either continuous or intermittent urinary incontinence since birth or purchase, with most patients under one year of age [2–4]. The cause of urinary incontinence in older patients may sometimes be a tumor of the urinary tract. Transition cell carcinoma is the most common tumor of urinary system in dogs [5]. Interestingly, in humans there are reports of metastatic lesions to the head and neck area from the urinary system [6]. Phenotypically, EUs can be either intramural or extramural, with intramural EUs identified in 64–95% of dogs [2, 7–9]. The incidence in female dogs are reportedly 20x more prevalent than males [2, 10]. Surgical correction of an ectopic ureter involves one of three techniques: ureteroneocystostomy, neoureterostomy, or nephroureterectomy. Ureteroneocystostomy requires the distal transection of the ectopic ureter with anastomosis of the proximal segment to the urinary bladder via an extravesicular, intravesicular, or combined technique [4, 11]. Neoureterostomy involves incising the bladder mucosa overlying the intramural portion of the ureter and suturing of ureteral mucosa the bladder mucosa. The ectopic segment distal to the new opening is then either ligated or resected to prevent persistent drainage through the ectopic submucosal tract [4, 11–13]. Nephrectomy is generally reserved for patients with non-functioning kidneys or intractable pyelonephritis who have normal function in the contralateral kidney [4]. When using the open surgical technique, both ureteroneocystostomy and neoureterostomy are associated with major abdominal and bladder trauma due to the wide surgical access [4]. Cystoscopy-guided laser ablation of intramural ectopic ureters is a minimally invasive treatment option, but requires specialized equipment (such as a laser) and appropriate training [14]. A procedure similar to cystoscopy-guided laser ablation is the recently described cystoscopic-guided scissor transection of intramural ectopic ureters [14]. Another minimally invasive surgical approach in animals is laparoscopic surgery, which has been used for many years in human ureteral transposition procedures [15–18]. This study aimed to determine the results of laparoscopic treatment of intramural ectopic ureters in female dogs. We hypothesized that laparoscopic ureteroneocystostomy is a safe and minimally invasive surgical approach for the treatment of ectopic ureters. This article presents the results of laparoscopic treatment of urinary incontinence, caused by ectopic ureters in female dogs. The laparoscopic technique presented is an alternative to cystoscopy techniques and surgery with laparotomy techniques for treatment of ectopic ureters.

## Materials and methods

### Inclusion criteria

The study population consisted of client-owned dogs that presented to the Department and Clinic of Surgery, Faculty of Veterinary Medicine, Wroclaw University of Environmental and Life Sciences with symptoms of urinary incontinence unresponsive to previous pharmacological treatments. All dogs with clinical signs of ectopic ureters were qualified for laparoscopic procedures. Clinical observations were carried out for a minimum of one year after surgery. The same operators, with experience in both laparoscopic and open surgeries, performed all procedures. This study was approved (written permission) by the Ethics Committee of the Wroclaw University of Environmental and Life Sciences (Faculty of Veterinary Medicine Animal Welfare Advisory Team No. 3.2023). All animal owners signed a consent form after being explained the details of the anesthesia and laparoscopic ureteroneocystostomy procedures and their associated risks.

### Diagnostic procedures

Before diagnostic imaging, complete blood tests (hematology and biochemistry), urinalysis, and urine cultures were performed. Diagnostic imaging, including native and contrast-enhanced abdominal computed tomography (CT) and genitourinary endoscopy, were performed during the same procedure. Ultrasonography was performed as a follow-up examination after surgery.

Complete blood analysis (hematology and biochemistry), urinalysis, and urine culture were performed before diagnostic imaging procedures. Diagnostic imaging examinations including abdominal computed tomography (CT) with and without contrast enhancement and urogenital endoscopy were performed during the same procedure. Ultrasonography was used as a control examination after surgery.

#### Computed tomography

Computed tomography (CT) of the abdominal cavity was performed using a 16-slice Siemens Somatom Emotion CT scanner. Scanning was performed along the long axis of the abdominal cavity, first in the cranial direction and then in the caudal direction. After performing a CT scan of the abdominal cavity, an intravenous contrast agent iomeprol (Prep. Iomeron 350 mg iodine/ml, Bracco) at a dose of 2 ml/kg.

#### Ultrasonography

Follow-up ultrasound was performed at 6, 12, 24, 48, and 72 h after surgery, followed by three consecutive examinations, each one week apart from the previous examination, using the Samsung RS 85 ultrasound machine with a linear probe (3–16 MHz) and a convex probe (4–9 MhZ).

### Urogenital endoscopy

**Fig 1:**
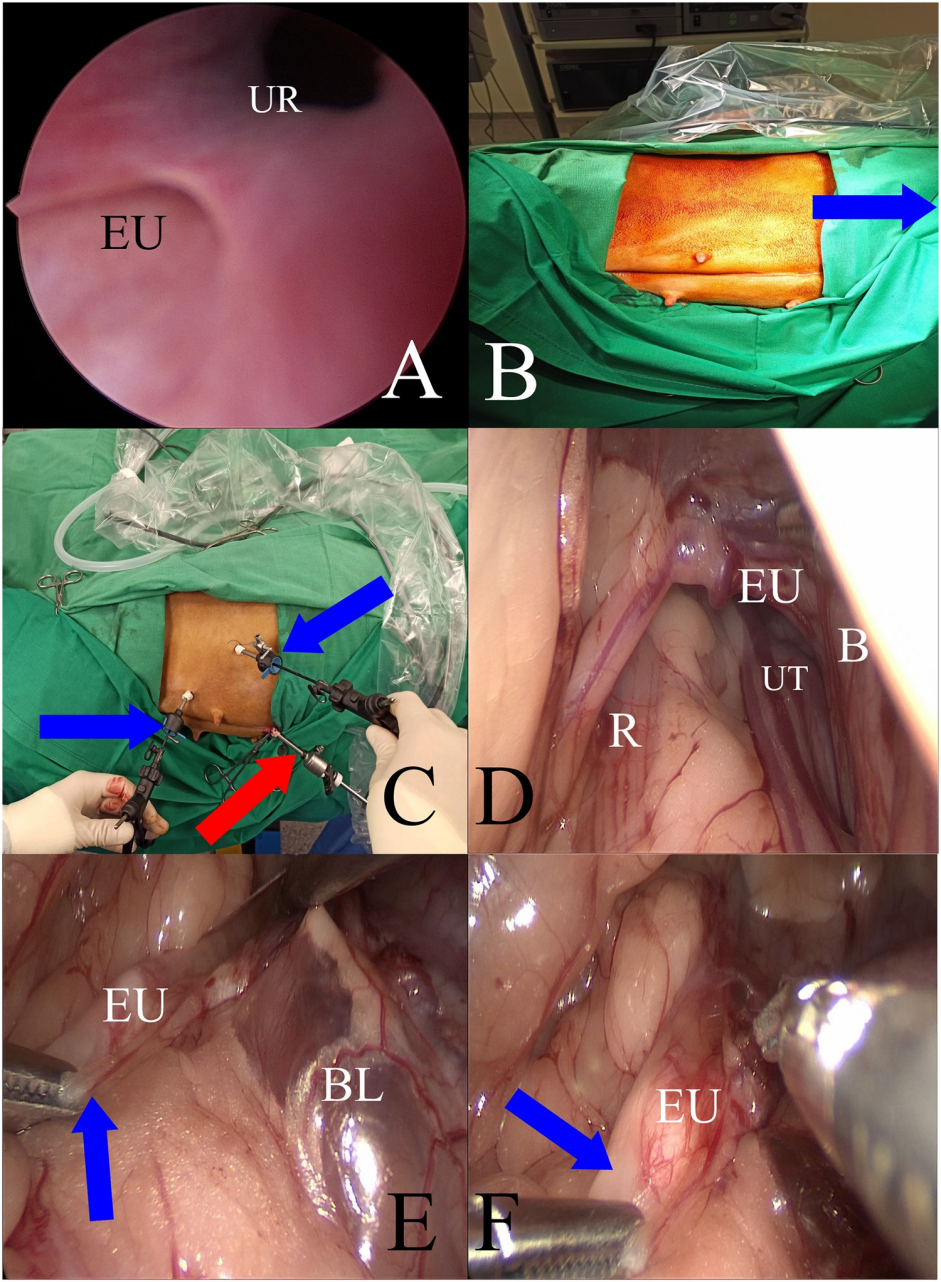
Cystoscopic assessment, patient positioning for surgery and stages of ectopic ureter preparation for laparoscopic ureteroneocystostomy: A: Cystoscopic view of an ectopic ureter (lower left corner) opening into the distal urethra (UR). B—Patient positioning for the laparoscopic ureteroneocystostomy in lateral recumbency with the affected (ectopic) side up;—the blue arrow points to the cranial part of the operated animal. C: Trocar placement during laparoscopic ureteroneocystostomy. Working trocars, blue arrows; optic trocar, red arrow. D: Laparoscopic intraoperative view of the ectopic ureter (EU) before dissection from the surrounding tissues. B, urinary bladder; UT, uterus; R, rectum. E, F: Laparoscopic operative view of the subsequent stages of ectopic ureter (EU) preparation from the surrounding tissues. BL: Bladder lateral ligament. During preparation, the grasp has to be of the tissues surrounding the ureter, not the ureter itself, to avoid damage. The blue arrow shows the correct way to hold the ureter when preparing.

After the induction of anesthesia, the dogs were positioned in dorsal recumbence for retrograde cystoscopy and vaginoscopy. The lower urinary tract was examined with a 2.7-mm or 4.0-mm rigid cystoscope with a passive flow of saline (0.9% NaCl) solution through the endoscope. Both ureteral orifices were identified and the reproductive and urinary tracts were evaluated for the presence of other abnormalities. Endoscopic examination of the genitourinary system was performed in each patient during the diagnosis of urinary incontinence due to ectopic ureters (Fig 1A) and additionally in three dogs one month after surgery because of persistent urinary incontinence symptoms.

### Anesthesia, pre-and postoperative management

The anesthesiologists found no contraindications to general anesthesia after conducting the clinical examination and considering the results of the blood tests. Patients were premedicated intramuscularly with a mixture of dexmedetomidine (Dexdomitor, Orion Pharma) at a dose of 5mcg/kg with methadone (Comfortan, Dechra) at a dose of 0.2 mg/kg. General anesthesia was induced with propofol (Scanofol, ScanVet) administered according to its effect (usually approximately 1 mg/kg). The patient was subsequently intubated and connected to a Mindray Wato Ex 65 inhalation anesthesia machine. Anesthesia was maintained using isoflurane (Isovet; Piramal Healthcare) in oxygen. Intraoperative analgesia was obtained by continuous infusion of fentanyl (Fentadon, Dechra) at 0.2 mcg/kg/min after previous bolus administration at 2.5 mcg/kg. Intravenous fluids (crystalloids) were infused at a rate of 5 ml/kg/h during anesthesia in all dogs. After transfer to the surgical area, the dogs were placed on a heating pad and instrumentation was initiated. Electrocardiography (ECG) using a computer-based ECG monitor was used to control the heart rate (HR) and arrhythmias. Systolic, diastolic, and mean blood pressure (SBP, DBP, and MBP, respectively) were measured noninvasively using the oscillometric method. The cuff was placed on the left or right forearm. The arterial oxygen saturation (SpO2) was monitored using a pulse oximeter placed on the tongue. End-tidal carbon dioxide (etCO2) and respiratory rate (RR) were measured using sidestream capnometry. The temperature was measured using an esophageal thermometer. Treatment of postoperative pain included the administration of buprenorphine (Bupaq Multidose, Orion Pharma) at a dose of 20 mcg/kg every 8h for the next 3 days and meloxicam (Metacam, Boehringer Ingelheim) for 3 days, initially at a dose of 0.2 mg/kg, then 0.1 mg/kg. In addition, on the first day, patients were given metamizole (Pyralgivet, Vet-Agro) at a dose of 25–50 mg/kg every 8 hours.

The operated dogs received antibiotics subcutaneously for 5 days. The first injection of amoxicillin-containing clavulanic acid (Synergal Inj. (140 + 35) mg/ml ScanVet Poland) at a dose of 12,5 mg/kg was administered just before surgery and repeated 24 h after surgery.

### Surgical procedure

**Fig 2:**
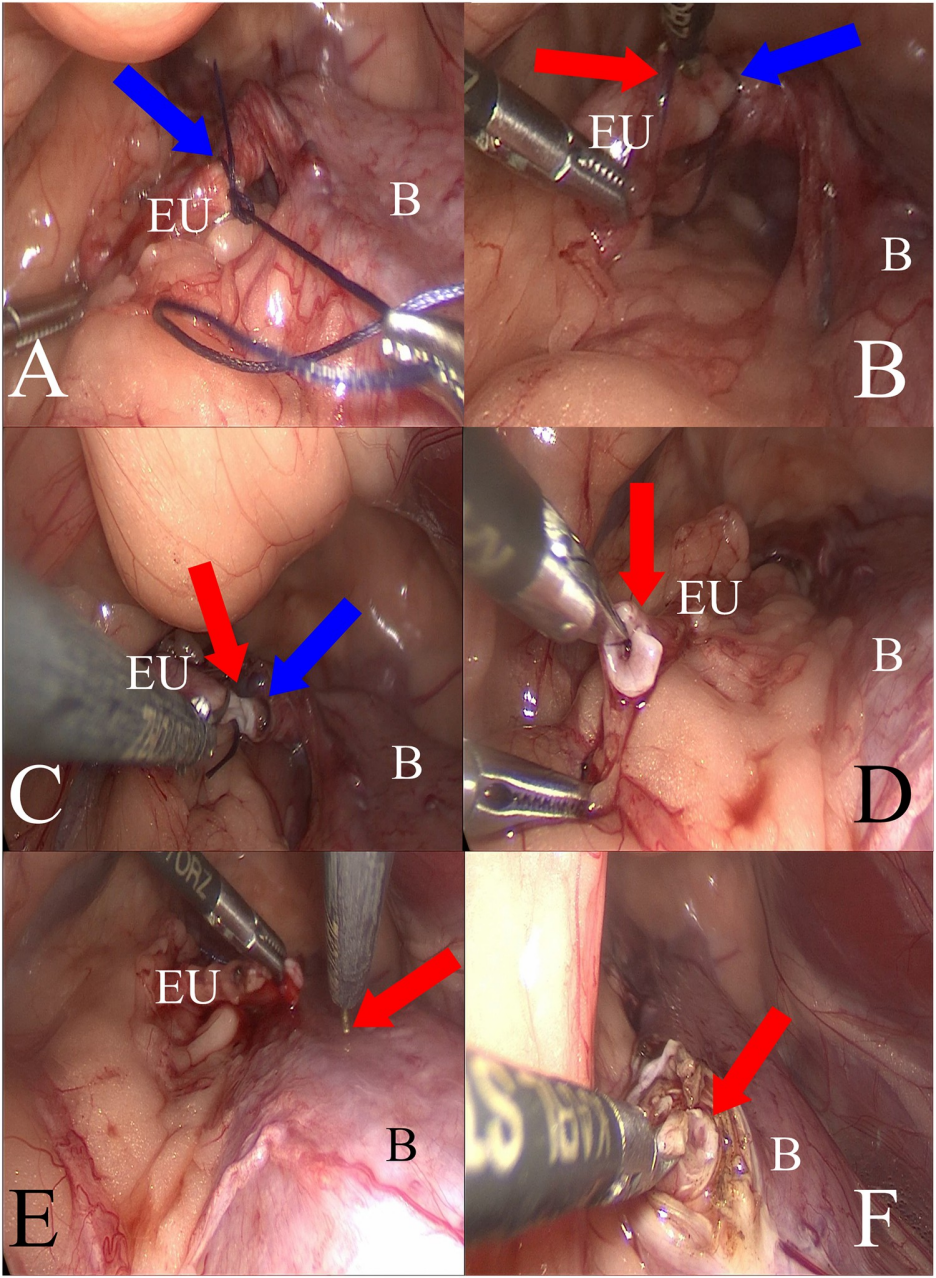
Laparoscopic intraoperative view of different stages of the ectopic ureter transposition: A—Ligation of the ectopic ureter (EU) and sliding knot (blue arrow); B, the urinary bladder. B—Red arrow indicates vessel coagulation on the surface of the ectopic ureter (EU) at the level of the planned transverse ureter incision. The blue arrow indicates the site of the previous ligation. B—urinary bladder. C: Transverse cut of the ureter (red arrow) near the previously placed ligation (blue arrow). EU: ectopic ureter. B: urinary bladder. D: Ureter is cut parallel to its long axis before transposition into the bladder lumen (red arrow); EU: ectopic ureter; B: urinary bladder. E—Placing the dissected and cut-off ectopic ureter (EU) to determine a spot for transposition to the bladder (B), which reduces the risk of excessive tension at the place of subsequent anastomosis. The red arrow indicates the laparoscopic hook and designated ureteral transposition site. F: Orifice in the bladder (B) wall of the transposed ureter (B). The diameter of the orifice was approximately equal to the outer diameter of the ureter. The red arrow indicates the bladder lumen after mucosal incision.

**Fig 3:**
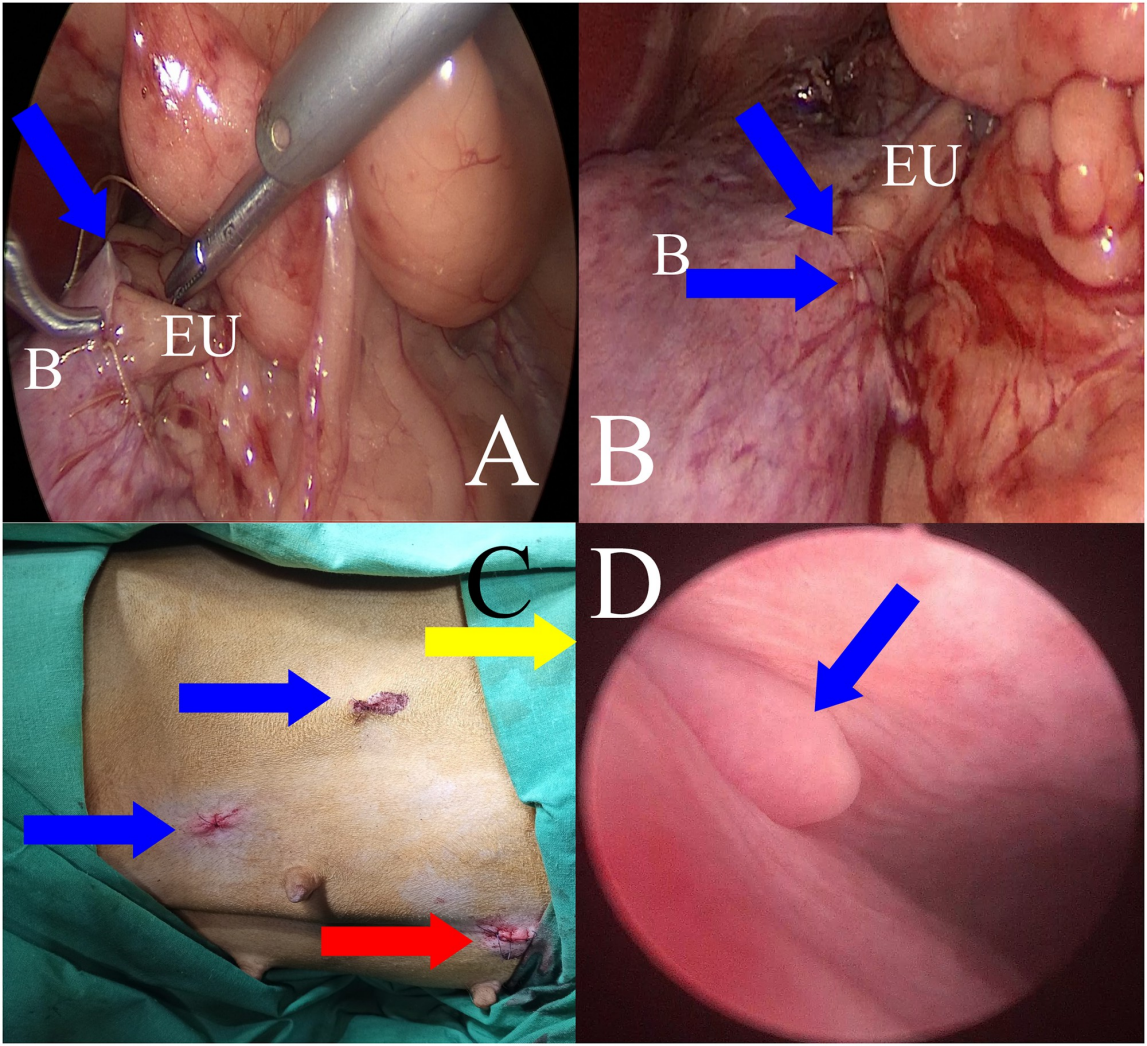
Laparoscopic intraoperative view of the anastomosis of the ureter with the bladder wall and the obtained postoperative result. A: Placing a suture between the ureter and bladder wall. Ectopic ureter (EU) after transposition into a new opening in the bladder (B). The blue arrow indicates the needle used during suturing. B—View of the performed anastomosis: blue arrows indicate the suture site. C: View of the postoperative wounds after the working trocars (blue arrows) and the optic trocar (red arrow); the yellow arrow indicates the cranial part of the patient. D—View of the transpositioned ureter (blue arrow) two months after surgery during control cystoscopy in a female dog with persistent urinary incontinence.

Before surgery, a Foley catheter was inserted into the bladder of each patient through which urine was removed, and sterile Ringer’s solution was introduced to slightly distend the bladder during surgery. The catheter was removed 24h or 48h after surgery. The patient was then placed in lateral recumbency with the affected (ectopic) side facing upward (Fig 1B). After antiseptic preparation in the surgical field, the patient was covered with a sterile surgical drape. The endoscopic equipment used for the laparoscopic procedure with a 5 mm 30° scope was manufactured by Karl Storz SE & Co. KG (Tuttlingen, Germany), and B Braun Aesculap (Tuttlingen, Germany). Pneumoperitoneum (CO_2_) was established using an open technique by introducing a 5-mm reusable trocar through a longitudinal incision in the umbilicus. The optics were inserted after reaching an insufflation pressure in the abdominal cavity, which was between 8 and 10 mmHg, depending on the patient’s size and adequate operative space. Subsequently, under the control of the endoscope, two consecutive 3,5 mm diameter trocars were inserted in a triangular fashion (Fig 1C). After gaining access to the abdominal cavity, an ectopic ureter was found (Fig 1D) and was gently dissected from the surrounding peritoneum and adipose tissue. (Fig 1E and 1F). Next, the ureter was ligated with suture material (polyfilament 2/0, Novosyn, B. Braun, Rubi, Spain) at the level of the bladder wall using the sliding knot technique (Fig 2A). Blood vessels were coagulated near the ligature to avoid bleeding (Fig 2B). Subsequently, the ureter was cut in the proximal part with laparoscopic scissors transverse to its long axis, leaving the ligated fragment in the urinary bladder (Fig 2C). The free ureteral fragment of the ureter was spatulated–3–5 mm from the transversely cut edge (Fig 2D). In the next stage, the procedure consisted of creating a new opening in the bladder wall proximal to the original orifice of the ectopic ureter with a laparoscopic hook. The site of the new opening was preselected by placing the dissected ureter in the planned transposition. This allowed us to choose the best location for the new orifice without excessive tissue tension at the anastomosis between the bladder and ureter walls (Fig 2E and 2F). After making a new hole in the bladder wall, a 4–5 mm long segment of the ureter was inserted into the bladder. Finally, the wall of the ureter was sutured to the bladder wall with 4–6 single stitches (polyfilament 5/0, Novosyn, B. Braun, Rubi, Spain), covering the serous and muscular layers of both organs, using the sliding knot technique (Fig 3A and 3B). When placing the suture through the ureteral wall, special care was taken not to occlude its lumen. Immediately after the sutures were placed, the surgical field was assessed to detect any bleeding or visible urine leakage at the anastomosis site. Placing the trocars in the manner described earlier allowed the procedure to be performed as planned, without the need to insert an additional trocar or convert to an open procedure. The trocar wound was closed using single intermittent sutures (monofilament 2/0; Dafilon; B. Braun, Rubi, Spain)—Fig 3C. The duration of all laparoscopic procedures was counted from the first incision to the last skin suture.

### Follow-up evaluation

A complete physical examination and abdominal ultrasound were recommended at 6, 12, 24, 48, and 72 h after the procedure, and then weekly for the first month after surgery to evaluate the morphology of the kidneys and ureters, as well as the site of ureterovesical anastomosis. No further rechecks were proposed if the dog regained full urinary continence. Additional checks for pharmacological management were scheduled for dogs with persistent or recurrent incontinence. If the dogs did not respond to the pharmacological treatment, cystoscopic urethral bulking was performed—Fig 3D.

At least one year after the operation, information about the health of all patients was obtained from the owners.

### Statistical analysis

The overall results were reported as median and range, and the numbers and percentages were reported for animals affected by the variables of interest. Available software (Microsoft Corp., Version 16.56, Microsoft, USA) was used for all the calculations.

## Results

Laparoscopic ureteroneocystostomy was performed on the first ten female dogs that were referred to our clinic with urinary incontinence, due to ectopic ureter. All dogs qualified for the procedure participated in observations for at least a year before the surgery. The median age at the time of laparoscopic ureteroneocystostomy was 13.5 months (range, 4 30 months). There were four Golden Retrievers: two American Staffordshire Terriers, one Entlebucher Mountain dog, one American pit bull terrier, one boxer, and one mixed breed. At the time of laparoscopic ureteroneocystostomy, three dogs had already undergone ovariohysterectomy (performed prior to referral), and the remaining seven were sexually intact. The median body weight at the time of laparoscopic ureteroneocystostomy was 24.4 kg (range, 10–35 kg). Urine cultures before surgery were negative in all patients, and all morphological and biochemical blood test results were within the reference ranges.

All dogs presenting with urinary incontinence had the severity of their state noted in their medical records, varying from urine leakage when resting or during excitement to constant dribbling of urine. The age of each dog when the first episode of incontinence occurred was known; urine leakage was noted by the breeder or after the owner purchased it.

Based on the CT and urogenital endoscopy results, intramural ureter ectopy was ascertained in every dog that qualified for surgery, of which six were left-sided and four were right-sided. All procedures were performed laparoscopically, without the need for conversion. Intraoperative localization of the ureter was possible thanks to the observation of its peristaltic movements. The mean operative time, measured from the first incision to the last suture, was 78.4 minutes, ranging from 61 to 95 min.

None of the dogs that underwent surgery experienced major early or distant complications. In three cases, leakage of blood-colored urine was observed from the catheter on the first day after surgery, and in one case, a trace of blood persisted until the fifth day after the catheter was removed. Follow-up ultrasound examinations performed 6 and 12 h after the procedure showed the presence of a small amount of free fluid around the bladder in four cases, which was no longer visible 24, 48, and 72 h after the procedures. No distant postoperative complications were found on follow-up ultrasound examinations performed weekly for one month after surgery. During the entire postoperative period, no leakage through the anastomosis or stenosis leading to a hydroureter or hydronephrosis was observed. The Post-operative wounds healed by primary intention. After catheter removal, on the first or second postoperative day, all operated dogs urinated on their own without any dysuria. Nevertheless, the two female dogs showed signs of urinary incontinence after surgery and did not respond to pharmacological treatment. Additionally, in one dog, stress-induced urinary incontinence was observed (in situations causing an increase in abdominal pressure). In these cases, the clinical symptoms of urinary incontinence temporarily resolved after urethral bulking with a urological preparation containing hyaluronic acid (Dexell^®^ VUR).

According to the information obtained from the owners, at least one year after the surgery, none of the dogs had urinary symptoms, including urinary incontinence or infection. The exceptions were three female dogs in which persistent urinary incontinence without concomitant infection was found after the first surgical treatment, and clinical improvement was observed after urethral bulking.

## Discussion

The results of the present study suggest that laparoscopic ureteroneocystostomy is a safe and effective alternative treatment of urinary incontinence due to ectopy of the ureters in female dogs. With this technique, more invasive open surgeries can be avoided in most cases. This applies especially to veterinary clinics where laparoscopic procedures are performed but without equipment for endourological procedures such as cystoscopy or lasers. Among the described in the literature major postoperative complications after EU correction can be mentioned: intestinal intussusception after neoureterostomy and uroabdomen after neocystoureterostomy [19]. None of them or other major postoperative complications were noted in our study. Noël et al. [20] reported that the major complication occurrence rate after open surgery EU correction that required surgical revision was 11% (5 dogs) and included uroabdomen (3 dogs) and severe dysuria due to colposuspension (2 dogs). According to Noël et al. [20], uroabdomen is secondary to leakage at the anastomotic site, dehiscence of the cystotomy incision, and leakage at the apex of the bladder (through the stay suture site). Among the minor complications in the three operated female dogs, traces of blood in the urine were described after the first 24 h after surgery. In only one case, slight coloration of the urine persisted for several days after the procedure. No minor complications such as pollakiuria, dysuria, or stranguria were found; nevertheless, hematuria occurred after surgery in 30% (3/10) of the cases. This value is more than twice lower than the incidence of the same complications after open EU surgery. Simultaneously, the percentage of minor complications (hematuria) after laparoscopic correction was approximately 17% higher than that after cystoscopy-guided laser ablation, with no other complications in cystoscopic EU correction [19]. As Dekerle et al. [19] showed that minor complications, including hematuria, accounted for 72% of all complications. At the same time lower urinary tract symptoms were more frequent after neoureterostomy (open surgery) compared to cystoscopic-guided laser ablation [19]. Clinical studies by Noël et al. [20] showed that the percentage of minor complications was 32% (15 dogs) when operated on using the open surgery technique and included dysuria (7; 47% of minor complications), pollakiuria (33%), and hematuria (20%). In a retrospective evaluation of cystoscopy-guided laser ablation of intramural ectopic ureters in female dogs, complications were observed in 32.2% of cases, including pollakiuria (16.1%), pigmenturia (9.7%), excessive vulvar licking (3.2%), and urinary tract infection (3.2%) [21].

Laparoscopic ureteroneocystostomy was feasible in 10/10 cases (100%) without the need for conversion. Jacobson et al. [14] showed in their work that they were able to completely transect the medial wall of the ectopic ureter in 7/8 of the operated cases, obtaining an anatomically correct position of the terminal ureteral orifice. However, Hooi et al. [21] in a retrospective review demonstrated that the UE could not be corrected in 9.7% of all cystoscopy-guided laser ablation cases owing to the presence of extramural EU or an extramural portion of the EU and bladder mucosa injury secondary to cystoscopy. It is the opinion of the authors of this article that although the study was performed only in female dogs with intramural ectopic ureters, the described laparoscopic technique will be applicable in cases of intra- or extramural EU in both sexes. Techniques for cystoscopic correction of EU have so far only been used in cases of intramural ectopic ureters in female dogs.

In the present study, 7/10 (70%) dogs regained urinary continence after laparoscopic correction of EU. The remaining 30% showed signs of urinary incontinence despite proper ureter correction (confirmed by cystoscopy) and no lower urinary tract infections. Ultimately, owing to the lack of reaction after pharmacological treatment, the aforementioned dogs regained urinary continence after urethral bulking with hyaluronic acid. In a study by Noël et al. [20], true recurrence of incontinence was observed in 35% of dogs during the study follow-up, and most dogs responded to adjuvant medical treatment. Postoperative continence rate after surgical correction of EU, depending on the report, ranges from 22% to 72% [8, 9, 12, 13, 22–25]. Interestingly, cystoscopy-guided laser ablation in female dogs has shown similar results, with urinary continence rates of 31% and 47% [3, 4]. However, after cystoscopy-guided scissor transection of intramural ectopic ureters, three of the seven dogs regained urinary continence as a result of the procedure alone [14, 26]. It is recognized that several factors may influence the persistence or recurrence of urinary incontinence after EU correction, such as lower urinary tract infection (UTI), recanalization of the ligated ureter, disturbed urethral closure due to residua intramural EU, congenital urethral sphincter mechanism incompetence (USMI), poorly developed trigone, hypoplastic bladder, vestibulovaginal stenosis, neurogenic abnormalities, hormonal imbalance or inadequate surgery [2, 7, 13, 23, 25]. In humans, prostatectomy procedures performed using minimally invasive surgery were beneficial in preserving erectile function compared with open retropubic radical prostatectomy, without a statistically significant difference regarding urinary incontinence or surgical margins [26].

It is worth emphasizing that the average time of the surgery itself was less than 80 min, and the time of individual procedures decreased with each procedure in the operated group, up to 61 min. The median duration of cystoscopy-guided scissor transection of intramural ectopic ureters was 105 min (range, 40–170 minutes) [14], whereas that of cystoscopy-guided laser ablation of the EU was 113 min (range, 57–280 minutes).

In the authors’ opinion, positioning dogs on their sides and using the three-trocar technique are good solutions. Owing to the obtained operating access, the entire planned procedure was unproblematic. However, it is not possible to compare these results because of the lack of veterinary literature describing laparoscopic ureteroneocystostomies.

No anastomotic leaks or stenoses were found in the dogs that underwent surgery. These complications are among the most serious after re-implantation of the ureter and have been described in both human and veterinary medicine [20, 27, 28]. Considering the possibility of the abovementioned complications, we decided to perform a series of regular ultrasound examinations, which allowed us to control the healing process of the ureterovesical anastomosis. In the operated group, a small amount of free fluid around four cases, during the control examination up to 12 h after the procedure. In our opinion, this could have been fluid coming out of the bladder during the transposition of the ureter, the volume of which did not require intraoperative suctioning. At the same time, subsequent ultrasound examinations showed no free fluid, which confirmed a tight anastomosis. The surgeons did not use ureteral stents during anastomosis, which can further reduce the risk of leakage or postsurgical stricture.

Many techniques of ureter reimplantation to the bladder wall have been described in the literature [29–32]. In the present study, we performed a serous-muscular anastomosis between the bladder wall and ureter, achieving very good clinical outcomes. This type of anastomosis is the described method of ureteral implantation in the technique of open surgery [33]. In addition, cystoscopy performed during urethral bulking did not reveal excessive granulation tissue around the stumps inserted into the lumen of the bladder. The lack of dilatation of the ureters and renal pelvis indicates that patency was maintained, even though ureteral stents were not used. Ureteral stents were very often used by surgeons to treat ureter stenosis [28], they are very practical and rarely associated with complications after surgical correction of the EU. However, lack of an appropriate stent may be a problem in small dogs EU surgery [34].

In the present study, we used an absorbable polyfilament material to suture the ureter to the urinary bladder wall. This was due to the simpler manipulation of the polyfilament material than that of the monofilament, which has been described and used in human laparoscopic surgery [33].

The important stage of the laparoscopic procedures is the proper anesthesia. Throughout our study, no anesthetic complications were observed either during or after surgery. The anesthesia method used and described in the study is, in the authors’ opinion, effective and safe for animals. The aforementioned safety is also an effect of medication used in preanesthesia, which was done with dexmedetomidine as sedative and adjuvant in anesthesia in different settings with reducing postoperative complications. Beneficial properties of dexmedetomidine were confirmed multiple times by different authors [35–37].

The main limitation of the present study was its small sample size and the lack of a control group for the long term outcomes. Ideally, all dogs would have had repeated cystoscopy to assess how the transplanted ureter healed and for any stenosis of the ureteral opening. However, given that most dogs improved and were doing well, this additional procedure, according to Jacobson et al. [14] report, was difficult to justify. Incontinence recurred in three dogs treated with laparoscopic ureteroneocystostomy, and cystoscopy was repeated during the urethral bulking injection. Urodynamic studies were not performed in any of the dogs in the present study, although these may provide useful information regarding the presence of any concurrent urethral sphincter mechanism incompetence or detrusor instability. Similar observations were made by other authors who did not conduct urodynamic studies in their patients suffering from ectopic ureters. However, it is a fact that no clear relationship was found between the measurement of maximal urethral closure strength by pre-operative urethral pressure profilometry and post-operative incontinence prognosis [12, 38, 39]. In the present study, we did not have an incontinence scale prepared based on postoperative questionnaires completed by owners, which may be problematic when comparing our results with those of other authors who had such scales [19, 21]. A certain limitation is the one-center study, which makes it impossible to compare the results of different centers and surgeons.

In the authors’ opinion, a difficulty resulting from minimally invasive techniques is the need for advanced skills in laparoscopic suturing. This intra-abdominal skill is considered one of the most challenging procedures in laparoscopic surgery [40]. Nevertheless, given the rapid development of veterinary minimally invasive surgery and the growing popularity of laparoscopic procedures, mastering endoscopic suturing skills should be in the interest of most, if not all, endoscopic surgeons.

In conclusion, laparoscopic ureteroneocystostomy appears to be a safe and effective minimally invasive surgery as an alternative to open surgery and cystoscopic laser ablation or cystoscopy-guided scissor transection for the treatment of urinary incontinence due to intramural ectopy of the ureters in female dogs.

The procedure presented in this publication can be used in facilities that do not have appropriate equipment or expertise in endourological ablation. Furthermore, larger studies are needed to confirm these results, and prospective comparative studies are important to determine any differences in long-term outcomes between the available treatment options for ectopic ureters. Studies on the effectiveness and safety of laparoscopic ureteroneocystostomy for the treatment of urinary incontinence due to extramural ectopy of the ureters in dogs would also be useful. Further inquiry should include also the comparison of laparoscopic ureteroneocystostomy with cystoscopic laser ablation, cystoscopy-guided scissor transection and open ureteroneocystostomy. This could help to indicate the least invasive and the most effective surgical technique in the treatment of urinary incontinence due to ectopic ureters. It would also be necessary to investigate the percentage of dogs that continue to show signs of urinary incontinence after surgery. The effectiveness of the laparoscopic ureteroneocystostomy should also be examined in the treatment of extramural ectopic ureters in female dogs as well as in both types of ectopic ureters in male dogs.

## Conclusions

Earlier suggestions that laparoscopic ureteroneocystostomy is a safe and minimally invasive surgical approach for the treatment of ectopic ureters were substantiated. Peristent signs of urinary incontinence in several of the operated female dogs were propably caused by other, undetected flaws of the urinary tract. The laparoscopic procedures were possible to conduct regardless of age and weight of the operated animals. Very good wound healing results and no postoperative complications allow us to recommend the described technique as the method of choice for surgical centers performing laparoscopic procedures. This recommendation includes not only intramural ectopic ureter but also an extramural type of ectopic ureter, where the cystoscopic technique is not available.
